# Use of a new silver barrier dressing, ALLEVYN^◊^ Ag in exuding chronic wounds

**DOI:** 10.1111/j.1742-481X.2009.00608.x

**Published:** 2009-06

**Authors:** Paula Kotz, Jane Fisher, Pat McCluskey, Samantha D Hartwell, Hussein Dharma

**Affiliations:** E T Consultant Services Inc.Asheville, NC, USA; P.G. H. Dip in wound healing, Wound Care, Cork University HospitalBishopstown, Cork, Ireland; Smith & Nephew Wound ManagementEngland, UK

**Keywords:** Bacterial barrier, Exudate management, Infection

## Abstract

Recognising and managing wounds at risk of infection is vital in wound management. ALLEVYN Ag dressings have been designed to manage exudate in chronic wounds that are at risk of infection; are displaying signs of local infection; or where a suspected increase in bacterial colonisation is delaying healing. They combine an absorbent silver sulfadiazine containing hydrocellular foam layer, with a perforated wound contact layer and highly breathable top film. The results presented are from a multi-centre clinical evaluation of 126 patients conducted to assess the performance of ALLEVYN Ag (Adhesive, Non Adhesive and Sacrum dressings) in a range of indications. Clinicians rated the dressings as acceptable for use in various wound types in 88% of patients. The majority of clinical signs of infection reduced between the initial and the final assessment. The condition of wound tissue and surrounding skin was observed to improve, and there was significant evidence of a reduction in the level of exudate from initial to final assessment (*p* < 0.001). Clinicians rated ALLEVYN Ag as satisfying or exceeding expectations in over 90% of patients. The evaluation showed the dressings to offer real benefits to patients and clinicians across multiple indications when used in conjunction with local protocols.

## INTRODUCTION

Infection in chronic wounds constitutes a major barrier to healing and can have an adverse impact on the patient's quality of life as well as on the healing rate of the wound. Infected wounds are likely to be more painful, hypersensitive and odorous, resulting in increased discomfort and inconvenience for the patient (1). In addition, infected wounds are often associated with higher exudate levels which can increase the number of dressing changes required; the amount of nursing time involved in managing them and the overall cost for the health care provider. Prolonged treatment of a wound because of infection is undesirable from the health care provider's as well as the patient's perspective and the timely use of an effective bacterial barrier dressing regime to manage suspected infection is a key aspect of wound management.

The negative effects of excess exudate on wound healing are well documented (2); this can result in delayed healing, maceration of peri-wound skin and embarrassing leakage for the patient (3). Numerous studies have, however, showed the benefits of maintaining a balanced moist environment at the wound interface, as this has been shown to facilitate closure and to speed up the healing process (4). It is important that modern wound dressings can maintain moisture balance by absorbing excess exudate and preventing the wound drying out, in addition to being able to afford bacterial barrier properties in wounds which are at risk of infection, when required.

Chronic wounds are contaminated with varying levels of bacterial flora, originating from the patients themselves or from the environment (5). These micro-organisms respond to environmental conditions and those in which they can survive at any one time depend on the chemical, physical and biological factors present.

The extent to which these bacteria affect the healing of the wound is influenced by the ability of the patients immune system to combat the bacteria (host resistance), the number of bacteria introduced (higher numbers are more likely to overcome host resistance) and the type of bacteria introduced. For example, some bacteria have greater disease-producing ability (virulence) than others and may be able to cause disease in relatively low numbers. Recognising wounds associated with clinical suspicion of infection or managing wounds at risk of developing infection is a subject of intense debate. Although all chronic wounds contain bacteria, often there is no detrimental effect. In contaminated wounds, the bacteria do not increase in number or cause clinical problems. Bacteria in colonised wounds will multiply, but will not damage the wound tissues. In an infected wound, the bacteria multiply, healing is disrupted and wound tissues are damaged (local infection). Bacteria may produce problems nearby (spreading infection) or cause systemic illness (systemic infection). Localised infection is often characterised by the classic signs and symptoms of inflammation, pain, heat, swelling, redness and loss of function. However, particularly in chronic wounds, bacteria may cause problems such as delayed (or stalled) healing, in the absence of such obvious indicators of inflammation. Some clinicians refer to this more subtle state of localised infection as ‘critical colonisation’, or ‘covert’ or ‘occult’ infection (1). Colonised wounds undoubtedly require vigilant monitoring and the timely use of dressings affording bacterial barrier properties as well as effective exudate management may be required where the patient is at risk of infection, displaying early signs of local infection or there is a clinical suspicion that increased bacterial bioburden is slowing progression to healing.

ALLEVYN Ag dressings, one of the most recent additions to the ALLEVYN hydrocellular dressing range, have been designed by Smith and Nephew to provide exudate management and bacterial barrier properties for chronic wounds that are at risk of infection, showing early signs of local infection or where increased bacterial bioburden is suspected to be delaying healing. ALLEVYN Ag dressings are available in adhesive, non adhesive, sacrum and heel format.

The dressings feature an advanced triple layered technology which combines an absorbent silver sulfadiazine containing hydrocellular foam layer, sandwiched between a perforated wound contact layer and a highly breathable top film. This unique ‘triple action technology’ is designed to absorb, retain and transpire the optimal balance of fluid so ensuring that a moist wound environment is maintained to facilitate wound closure while minimising the risk of peri-wound skin maceration or leakage occurring. Excess exudate is absorbed into the hydrocellular foam core in a rapid yet controlled fashion upon application of the dressing. Once exudate approaches the top of the dressing, it evaporates through the highly breathable film layer. This upper film layer had been designed to respond to the presence of excess exudate within the dressing by increasing its moisture vapour transmission rate. This allows water vapour to be released at an increased rate so ensuring that the dressing does not become saturated but remains comfortable and conformable for the patient to wear. The film layer is, in addition, a waterproof bacterial barrier which prevents potential extrinsic contamination of the wound.

The silver compound, silver sulfadiazine, incorporated within the dressing was introduced into clinical practice in 1968 and has been safely used for over half a century as a topical antimicrobial agent in burns and other wound types as it is well documented to be effective against a broad spectrum of common wound pathogens (6–9). Silver is known to kill pathogens in a variety of ways by deactivating metabolic pathways and genetic machinery and by weakening bacterial cell walls and in vitro testing has showed ALLEVYN Ag to have bacterial barrier characteristics to a variety of bacterial strains.

The combination of excellent exudate management and bacterial barrier properties protection afforded by ALLEVYN Ag helps to overcome two major barriers to wound healing and promotes an environment supportive of granulation and re-epithelialisation while preventing peri-wound maceration.

The aim of this evaluation was to generate data on the performance of ALLEVYN Ag adhesive, ALLEVYN Ag Non Adhesive and ALLEVYN Ag Sacrum dressings in a clinical setting on multiple indications.

This was a non comparative clinical evaluation conducted across six countries with a total of 24 participating centres.

## METHODS

A multi-centre clinical evaluation (using a Case report form as data capture on the product in use for up to six dressing changes) was performed between October 2007 and March 2008.

A total of 126 patients were recruited from the adult (≥ 18 years) populations routinely seen by the evaluation clinicians from across the UK, Spain, Ireland, France, Germany and USA. Ethics review of the study documentation was not sought prior to the data collection as the evaluation involved no change to patient treatment. The product is available within the countries involved. There were no patient identifiers (name, date of birth, etc) in the study data capture, and therefore the study did not require review by a research ethics committee. Institutional approval was obtained if required.

The patients recruited were suitable to the product in accordance with the indications and contra-indications in the standard product insert leaflet and were treated according to the insert leaflet's instructions for use and standard centre practice throughout the evaluation. Consent was given by patients prior to participation using the centre's own consent forms which also included consent for any photographs taken. The evaluation was in the form of a collection of case studies documented using a standard data collection form (provided by Smith & Nephew), which allowed the data gathered to be pooled and summarised to provide an understanding of the uses and performance of the products in a clinical setting on multiple indications.

No additional procedures, other than completion of the data collection form and photography of the wound (if appropriate and with the patients consent), were performed during the patient's participation in this evaluation. Additional restrictions were not placed on the patient or on their concomitant medication/therapies as a result of taking part in the evaluation.

The following inclusion criteria were specified: males or females (not pregnant or lactating) of at least 18 years of age; patients who were able to understand and were willing to consent to the evaluation, and patients who had an exuding wound present.

If the patient met any of the following criteria, then they were excluded from the evaluation: patients with a known history of poor compliance with medical treatment; patients who have participated in this evaluation previously and who healed or were withdrawn. Other exclusion criteria were patients with known sensitivity to silver sulfadiazine or other product components; patients with clinically infected wounds requiring systemic antibiotics and patients with wounds requiring the use of any other topical antimicrobial agent on the wound.

The study objectives were to determine whether the dressings were acceptable to clinicians for their indicated uses (primary) and to assess dressing performance parameters. These were as follows: durability (wear time), whether the dressing was easy to apply and remove; pain on application and removal; reasons for removal of the dressing; trauma to the wound and surrounding skin on removal; adherence to the wound/surrounding skin (adhesive dressings); patient comfort during wear; conformability; satisfaction with exudate management and leakage, and appearance of the skin (discolouration). Secondary objectives included assessing changes in the wound over the course of treatment (maximum of six dressing changes) against the following parameters:

- Clinical signs of infection and whether the wound was clinically infected
- Wound size
- Wound appearance (tissue types)
- Appearance of surrounding skin
- Exudate level

Secondary objectives also included determining whether the clinicians were sufficiently satisfied with the dressing's overall product performance in terms of exudate management, bacterial barrier properties (US centres) or antimicrobial properties (European centres), general ease of use, durability, patient comfort and convenience.

## STATISTICAL METHODS

Wilcoxon's Signed Ranks test was used to test for a difference in the wound area from initial to final assessment. Cochran–Mantel–Haenszel test stratified by patient was used to test for a difference in the level of exudate between baseline and final assessment. McNemar's test was used to test for a difference in the percentage of patients presenting with any clinical signs of infection at the baseline and final assessment. All statistical tests were two-sided and conducted at the 5% significance level.

## RESULTS

### Patient characteristics

A total of 126 patients were evaluated from 24 centres (16 USA, 8 EU) during the period September 2007–March 2008. The mean age of patients was 67.8 years (range of 19–99 years) with a gender distribution of 59 (46.8%) males and 67 (53.2%) females. Patients were treated in a variety of settings including wound clinics (58; 46%), hospitals (26; 20.6%), patient's homes (17; 13.5%), nursing homes (16; 12.7%) and in a combination of hospital and patient homes (1; 0.8%) and hospital and nursing homes (1; 0.8%). The remaining seven (5.6%) were treated in other treatment settings, including medical practices for vascular diseases, long stay centres or nurse practitioner's offices. The median duration in the study for all patients was 21 days. At baseline, the median reference wound duration was 8.7 weeks (range 0.1 weeks to 14 years). Level of pain from the reference wound was recorded on a visual analogue scale (VAS) with 0 and 10 representing no pain and worst pain, respectively. The majority of patients (84; 68.3%) had no pain from the reference wound with a mean pain score of 0.9 (range 0–9.0). The majority of patients had the reference wound located on the lower leg (61; 48.4%), foot (21; 16.7%) or ankle (14; 11.1%), with the remainder distributed between anterior trunk (11; 8.7%), sacrum (7; 5.6%), buttocks (4; 3.2%), head/neck (2; 1.6%), arm (2; 1.6%), hand (2; 1.6%) and upper leg (2; 1.6%).

### Dressing applications and wound types

The patient wound types receiving ALLEVYN Ag dressings are summarised in Table 1.

**Table 1:** Patient wound types

Wound type	*n*	%
Venous leg ulcer	37	29.4
Surgical/graft	24	19.0
Pressure ulcer	20	15.9
Mixed/arterial leg ulcer	12	9.5
Traumatic	11	8.7
Diabetic foot ulcer	8	6.3
Burn	7	5.6
Other/unknown	7	5.6

#### Primary objective

Clinicians assessed the overall acceptability for 111 patients. The dressings were rated as acceptable for the majority of patients (98; 88.3%) and wound types.

#### Secondary objectives

##### Overall level of satisfaction with the evaluation dressing

**Figure 1:**
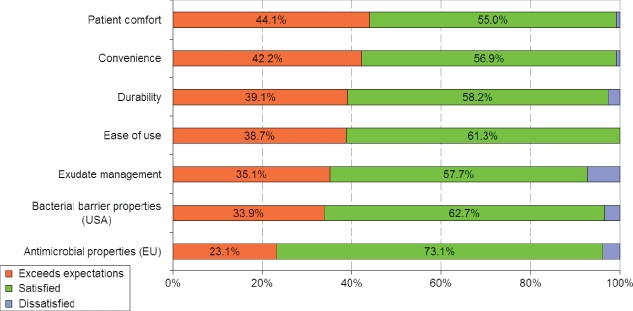
Overall satisfaction with the dressings.

For the majority of patients, clinicians judged the evaluation dressings as satisfactory or exceeding expectations with regards to exudate management, bacterial barrier properties (US centres), antimicrobial properties (EU centres), ease of use, durability, patient comfort and convenience (Figure 1).

### Clinical signs of infection

**Figure 2:**
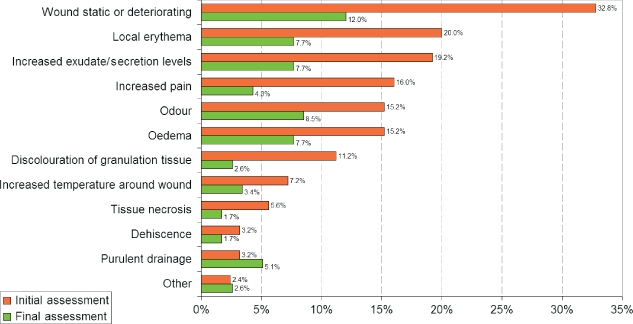
Percentages of patients exhibiting clinical signs of infection.

There was significant evidence of a reduction in the percentage of patients presenting with any clinical signs of infection between the initial and the final assessment (*p* < 0.001); corresponding to 79 (68.1%) patients presenting with clinical signs of infection at the initial assessment reducing to 36 (31.0%) at the final assessment (116 patients assessed at both the initial and the final assessment). Furthermore, a total of only ten (8.5%) patients were judged to have a clinically infected wound at the final assessment (118 patients assessed at both the initial and the final assessment). A reduction in the majority of clinical signs of infection between the initial and the final assessment was also observed (Figure 2).

### Wound size

There was significant evidence of a reduction in the wound area from the initial to final assessment (median reduction = 61%, *p* < 0.001). At the initial assessment, the median wound area was 6.2 cm^2^, reducing to 1.3 cm^2^ at the final assessment. At the end of the evaluation, 34 (27%) patients achieved complete wound closure.

### Level of exudate

**Figure 3:**
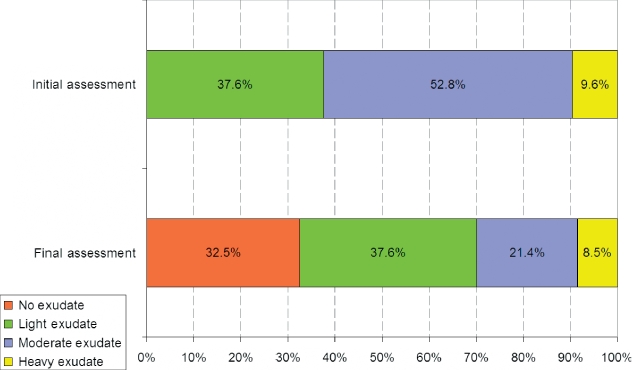
Wound exudate levels at the initial assessment and final assessment.

There was significant evidence of a reduction in the level of exudate from initial to final assessment (*p* < 0.001) (Figure 3).

### Appearance of the reference wound

Overall, the percentage of devitalised tissue (of the 89 patients who presented with devitalised tissue) was observed to have reduced between the initial and the final assessment. Furthermore, over all patients, there was an increase in healthy tissue at the final assessment (Table 2).

**Table 2:** Percentage of tissue types in wound bed

	Initial assessment		Final assessment	
	Mean %	Median %	Mean %	Median %
Pink (epithelial) tissue	9.5	0	44.8	30
Beefy red granulation	26.4	10	31.3	12.5
Dull red tissue	13.2	0	7.6	0
Friable granulation tissue	12.6	0	6.7	0
Yellow sloughy tissue	29.6	20	15.3	0
Black necrotic tissue	2.2	0	1.8	0
Devitalised tissue (patients with devitalised tissue at baseline)	44.1	50.0	21.4	10.0
Devitalised tissue (patients without devitalised tissue at baseline)	0	0	5.5	0

### Condition of the surrounding skin

**Figure 4:**
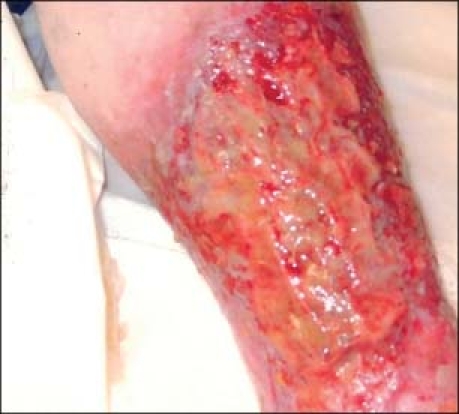
A 65-year-old female with a venous leg ulcer that had been present for 8 weeks. At initial assessment, the wound was heavily exuding, covered with sloughy tissue, surrounded by erythema and malodorous.

**Figure 5:**
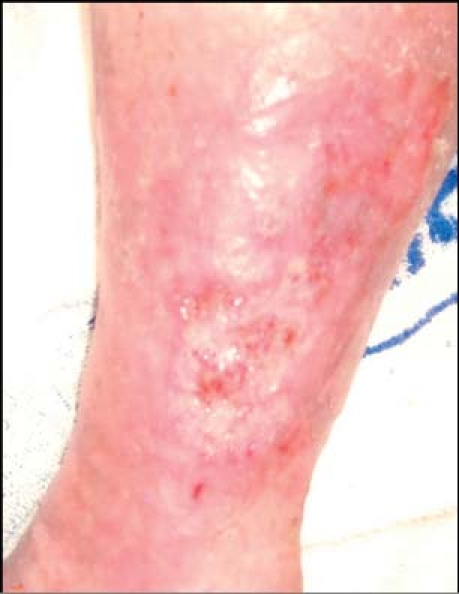
At final assessment after ALLEVYN Ag non adhesive was used in conjunction with compression and by the final dressing change, the ulcer was progressing towards closure with 90% of the wound epithelialised.

The percentage of patients with healthy skin was observed to have increased from the initial to final assessment, while the percentage of patients with unhealthy skin conditions was observed to have decreased at the final assessment (Table 3). Figures 4 and 5 show the improvement in one patient.

**Table 3:** Condition of the surrounding skin

	Initial assessment		Final assessment	
	*n*	%	*n*	%
Healthy skin	43	34.1	69	58.5
Inflamed	40	31.7	19	16.1
Macerated	27	21.4	9	7.6
Dry and flaky	20	15.9	7	5.9
Other	20	15.9	11	9.3

### Product performance parameters

Table 4 shows a summary of the product performance across dressings.

**Table 4:** Product performance parameters

Product performance	Percentage of
parameter	dressing changes
Dressing easy to apply	98.0%
Dressing easy to remove	99.2%
Comfortable to wear	97.7%
Good/acceptable dressing conformability	
On application	99.8%
During wear	99.6%
No discolouration to surrounding skin	67.8%
Slight discolouration to surrounding skin	24.8%
No pain on dressing application	80.4%
No pain on dressing removal	78.6%
Adhesive dressings fully adhered	
On application	91.6%
Prior to removal	76.8%
Satisfied with exudate and leakage handling of dressing	95.2%
Dressings changed for routine reasons	90.8%
	Mean (min, max)
Wear time (overall patients)	3.8 days (range 1–7.2)

### Safety

Of the 659 ALLEVYN Ag dressing applications applied to 126 patients, there were only five product complaints over the duration of the evaluation (0.8% of dressing applications). These were as follows: redness/irritation (one instance), erythema/excoriation (one instance), dermatitits (one instance), allergic reaction (one instance) and wound edge discolouration (one instance). These kinds of complaints are not unusual given the type of dressing and wounds being evaluated. This equates to a low level of product complaints in terms of exposure and suggests no concerns with the safety of the evaluation products.

## DISCUSSION

For the majority of patients (88%), ALLEVYN Ag was rated as acceptable across the indications treated. Overall, the average wear time of the dressings was 3.8 days (mean patient wear time), which may be attributed to the dressing being changed for routine reasons in the majority of cases.

The condition of the patients surrounding skin was observed to improve over the duration of treatment, with the percentage of patients with healthy skin increasing and all other unhealthy skin conditions decreasing at the final assessment compared to the initial assessment.

The percentage of patients presenting with clinical signs of infection present in the wound and the level of exudate from initial to final assessment were shown to decrease, both of which are a desirable outcome in terms of the patient and health care provider, and shows the value of these dressings to manage exuding chronic wounds with the potential for infection when used in conjunction with local protocols.

## CONCLUSIONS

Product performance

- For the primary objective of the study, the clinicians rated the dressing as acceptable in 88% of the patients included in the evaluation.
- For the secondary objectives, the majority of clinicians judged the dressings to be satisfactory or exceeding expectations with regard to exudate management, bacterial barrier properties, ease of use, durability (wear time), patient comfort and convenience.

Clinical performance

- There was significant evidence of a reduction in the percentage of patients presenting with clinical signs of infection at the initial and final assessment.

Wound characteristics

- There was significant evidence of a reduction in the wound area from initial to final assessment.
- Twenty-seven percent of patients had complete closure of their wounds during a median study period of 21 days.
- There was significant evidence of a reduction in the level of exudate from initial to final assessment.
- The extent of devitalised tissue in the wound was observed to reduce overall with a higher percentage of healthy tissue in the wounds over the course of up to six dressing changes.
- The condition of the surrounding skin was observed to improve over the course of up to six dressing changes.

ALLEVYN Ag is a bacterial barrier dressing that offers the following:

- Optimal exudate management leading to reduced maceration.
- Active and sustained reduction in microbial colonisation within the dressing in chronic wounds allowing the host to regain control and progress the wound to healing.
- A convenient choice for the management of critically colonised or infected chronic wounds when used in combination with local clinical protocols.

◊ALLEVYN is a trademark of Smith & Nephew
